# Supplementary material for: Weakening of the AMOC and strengthening of Labrador Sea deep convection in response to external freshwater forcing
Source: Nat Commun. 2024 Nov 28;15:10357. doi: 10.1038/s41467-024-54756-3 (PMC11605065; doi:10.1038/s41467-024-54756-3)
Supplement: Supplementary file 1 — Supplementary Information [file 41467_2024_54756_MOESM1_ESM.pdf]

Supplementary Information for

Weakening of the AMOC and Strengthening of Labrador Sea Deep Convection in Response to External Freshwater Forcing

Xinyue Wei[1] and Rong Zhang[2,1]

[1] Program in Atmospheric and Oceanic Sciences, Princeton University, Princeton, NJ, USA
[2] NOAA/OAR/GFDL, Princeton, NJ, USA

Contents of this file

Supplementary Figure 1. The anomaly of the density-space Atlantic Meridional Overturning Circulation (AMOC) streamfunction (Sv, $1\,Sv = 10^6\,m^3\,s^{-1}$) (water hosing - control)

Supplementary Figure 2. The extra-tropical North Atlantic subsurface (413m) temperature anomaly (water hosing – control)

Supplementary Figure 3. The climatological mean and anomalies of Atlantic Meridional Overturning Circulation (AMOC) streamfunction, surface forced water mass transformation (WMT_S) and interior mixing forced water mass transformation (WMT_M)

Supplementary Figure 4. The climatological mean of velocity ($m\,s^{-1}$) across the Overturning in the Subpolar North Atlantic Program (OSNAP) section in the control simulation

Supplementary Figure 5. The transient evolution of anomalous water properties across the Overturning in the Subpolar North Atlantic Program (OSNAP) section

Supplementary Figure 6. Comparison of the transient salt-based freshwater fraction (FWF) anomalies (FWF'_{salt}) and dye-based FWF anomalies (FWF'_{dye}) at the depth of 625m

Supplementary Figure 7. Comparison of salt-based freshwater fraction (FWF) anomalies (FWF'_{salt}) and dye-based FWF anomalies (FWF'_{dye}) across the Overturning in the Subpolar North Atlantic Program (OSNAP) section

Supplementary Figure 8. Relationship between the Labrador Sea vertical density difference and mixed layer depth (MLD) anomalies

Supplementary Figure 9. Comparing the model used in this study with Coupled Model Intercomparison Project Phase 6 (CMIP6) models for representing the Iceland-Scotland Overflow Water (ISOW)-associated Northeast Atlantic Deep Water (NEADW) layer in the deep Labrador Sea

Supplementary Figure 10. Comparison of the mean Atlantic Meridional Overturning Circulation (AMOC) streamfunction (Sv, $1\,Sv = 10^6\,m^3\,s^{-1}$) across the Overturning in the Subpolar North Atlantic Program (OSNAP) section in the control simulation with OSNAP observations in density space

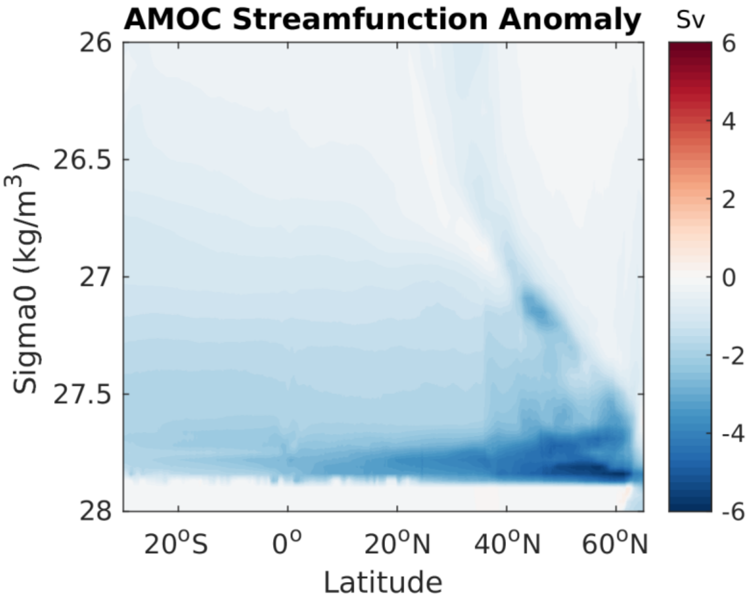

Supplementary Figure 1. The anomaly of the density-space Atlantic Meridional Overturning Circulation (AMOC) streamfunction (Sv, $1\ Sv = 10^6\ m^3\ s^{-1}$) (water hosing - control). The last 40-year average is used.

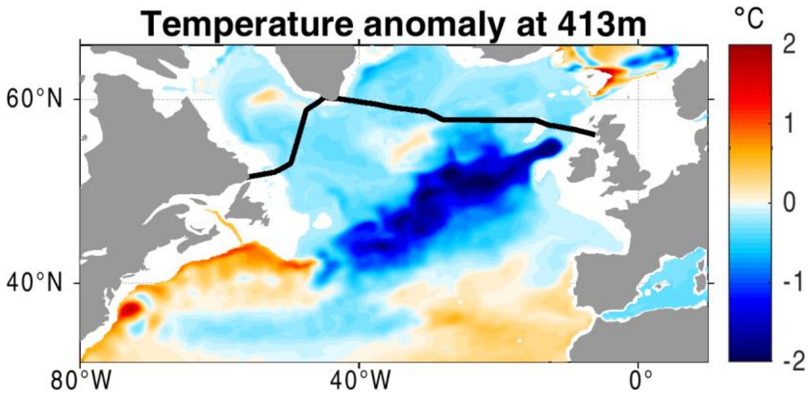

Supplementary Figure 2. The extra-tropical North Atlantic subsurface (413m) temperature anomaly (water hosing – control). The last 40-year average is used.

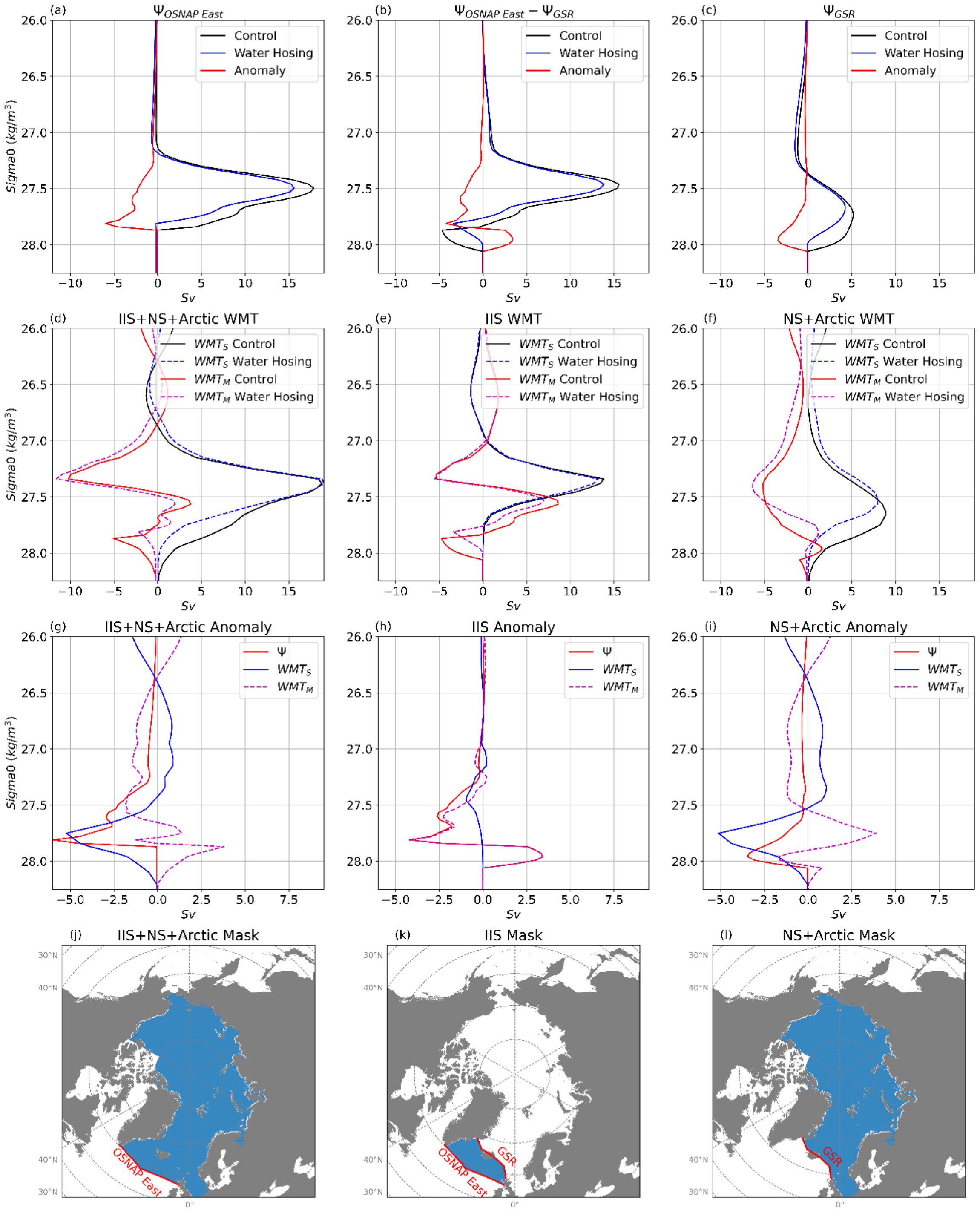

Supplementary Figure 3. The climatological mean and anomalies of Atlantic Meridional Overturning Circulation (AMOC) streamfunction, surface forced water mass transformation ($\mathrm{WMT_S}$) and interior mixing forced water mass transformation ($\mathrm{WMT_M}$). (a) The AMOC streamfunction across Overturning in the Subpolar North Atlantic Program (OSNAP) East. (b) The AMOC divergence (i.e. difference of the AMOC streamfuctions) between OSNAP East and Greenland-Scotland Ridge (GSR). (c) The AMOC streamfunction across GSR. (a-c) Black solid: control simulation. Blue solid: water

hosing experiment. Red solid: anomalies (water hosing – control). (d-f) Surface and interior mixing forced water mass transformation (WMT_S and WMT_M) over the region northeast of OSNAP East (d), between OSNAP East and GSR (i.e. Iceland-Irminger Seas (IIS)) (e), and northeast of GSR (f). Black solid: WMT_S in the control simulation. Blue dashed: WMT_S in the water hosing experiment. Red solid: WMT_M in the control simulation. Meganta dashed: WMT_M in the water hosing experiment. (g-i) The anomalies (water hosing – control) of the AMOC streamfunction (red solid), WMT_S (blue solid), and WMT_M (meganta dashed) over the region northeast of OSNAP East (g), between OSNAP East and GSR (i.e. IIS) (h), and northeast of GSR (i). (j-l) The mask for the region northeast of OSNAP East (including IIS, Nordic Sea (NS) and Arctic) (j), between OSNAP East and GSR, i.e. IIS (k), and northeast of GSR (including NS and Arctic) (l). The last 40-year average is used.

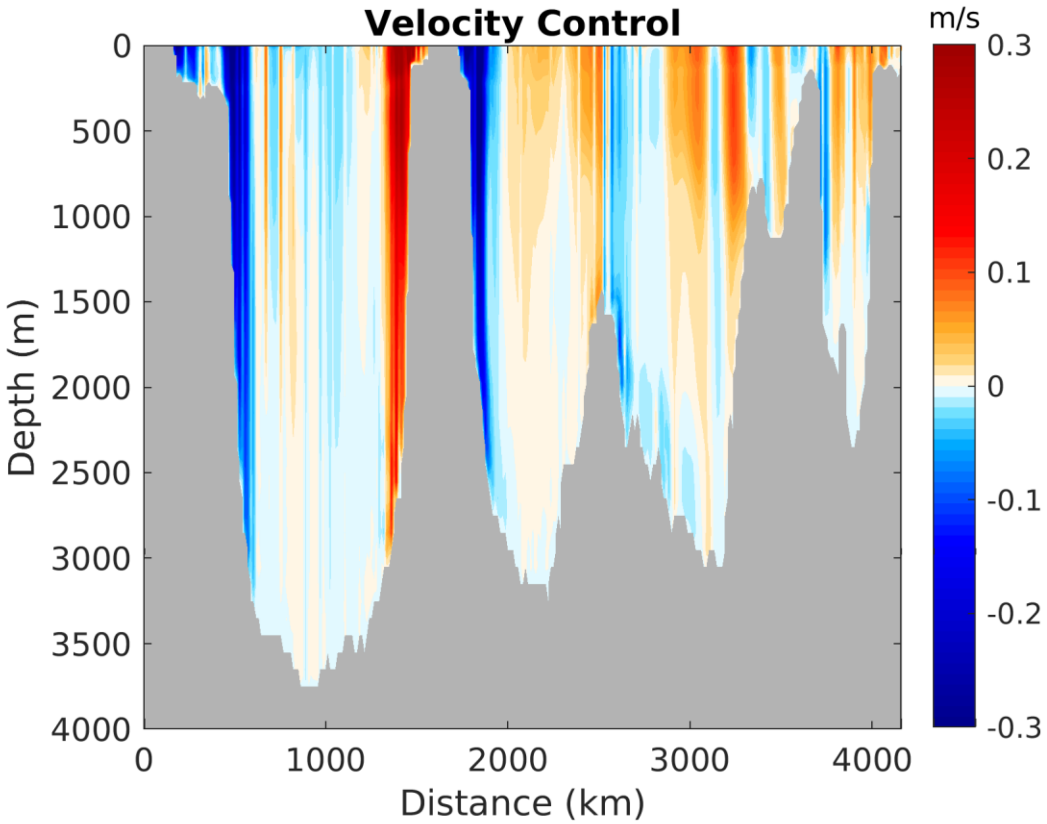

Supplementary Figure 4. The climatological mean of velocity ($m\,s^{-1}$) across the Overturning in the Subpolar North Atlantic Program (OSNAP) section in the control simulation. The last 40-year average is used.

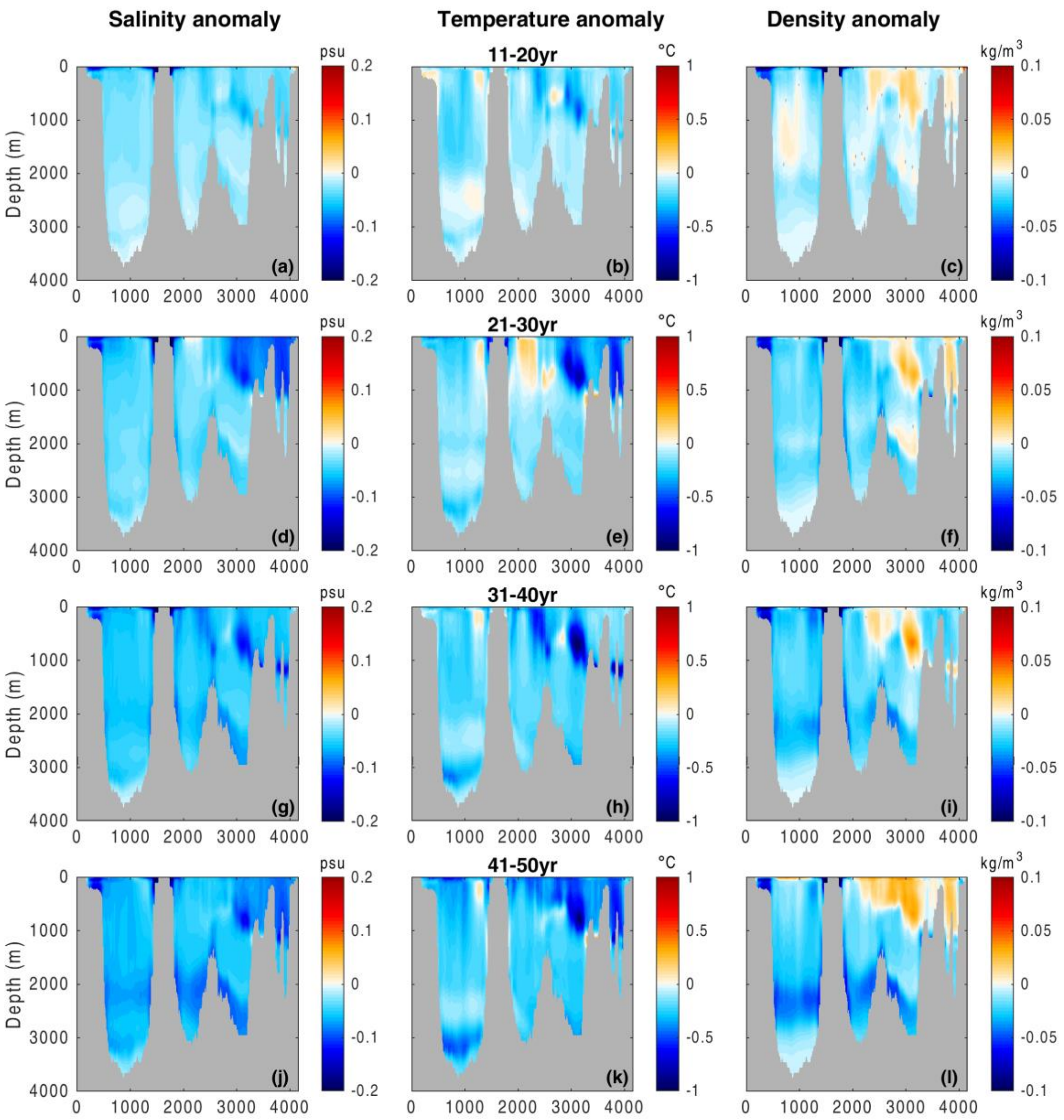

Supplementary Figure 5. The transient evolution of anomalous water properties across the Overturning in the Subpolar North Atlantic Program (OSNAP) section. The transient salinity anomalies (psu; a, d, g, j), potential temperature anomalies ($\circ C$; b, e, h, k), and potential density anomalies ($kg\ m^{-3}$; c, f, i, l) across the OSNAP section. (a-c) Averaged over years 11-20. (d-f) Averaged over years 21-30. (g-i) Averaged over years 31-40. (j-l) Averaged over years 41-50.

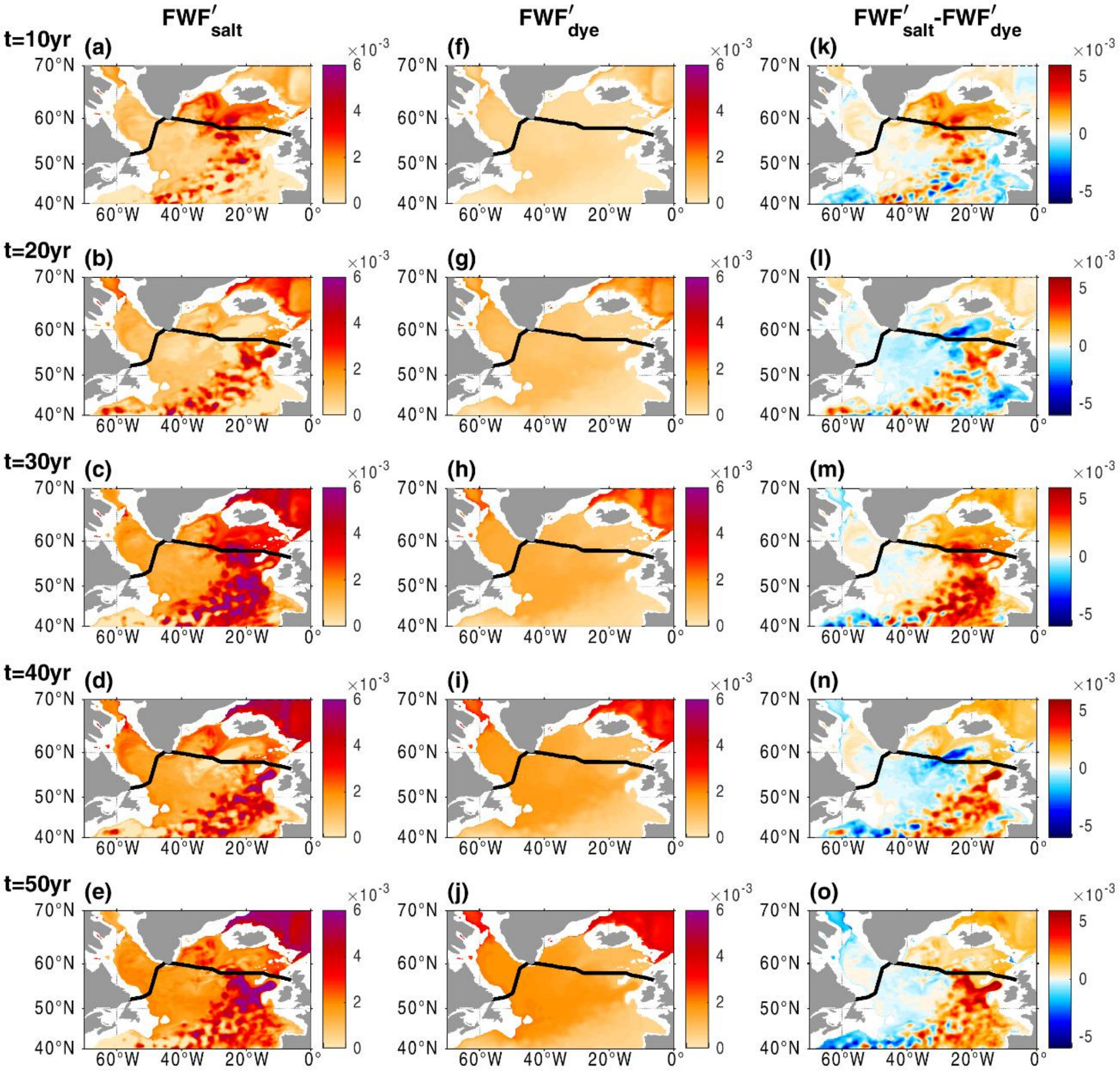

Supplementary Figure 6. Comparison of the transient salt-based freshwater fraction (FWF) anomalies (FWF'_{salt}) and dye-based FWF anomalies (FWF'_{dye}) at the depth of 625m. The horizontal maps show FWF'_{salt} (a-e), FWF'_{dye} (f-j), and their differences $FWF'_{salt} - FWF'_{dye}$ (k-o) at the depth of 625m at different years. **(**a, f, k) At year 10. (b, g, l) At year 20. (c, h, m) At year 30. (d, i, n) At year 40. (e, j, o) At year 50. This figure is similar to Fig. 6, 7, but is at 625m and extended to 70°N to include the Nordic Sea overflow pathways across the Greenland-Scotland Ridge.

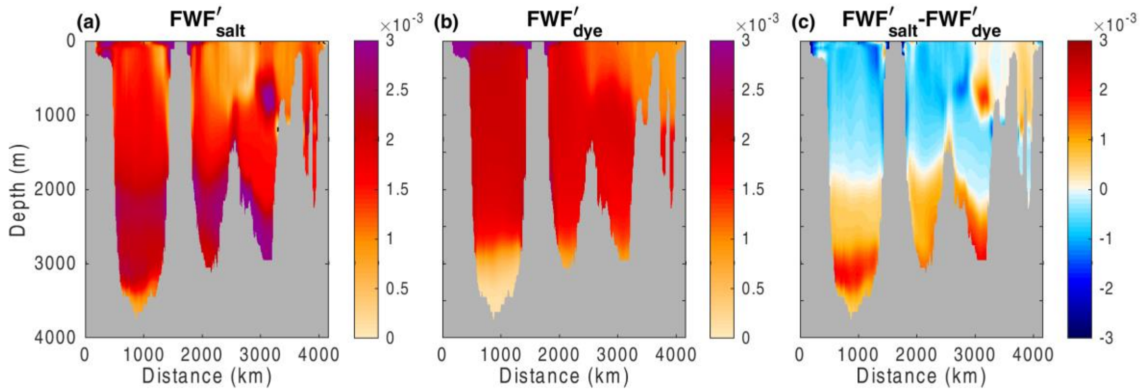

Supplementary Figure 7. Comparison of salt-based freshwater fraction (FWF) anomalies (FWF'_{salt}) and dye-based FWF anomalies (FWF'_{dye}) across the Overturning in the Subpolar North Atlantic Program (OSNAP) section. (a)FWF'_{salt}. (b) FWF'_{dye}. (c)$FWF'_{salt} - FWF'_{dye}$. The last 40-year average is used.

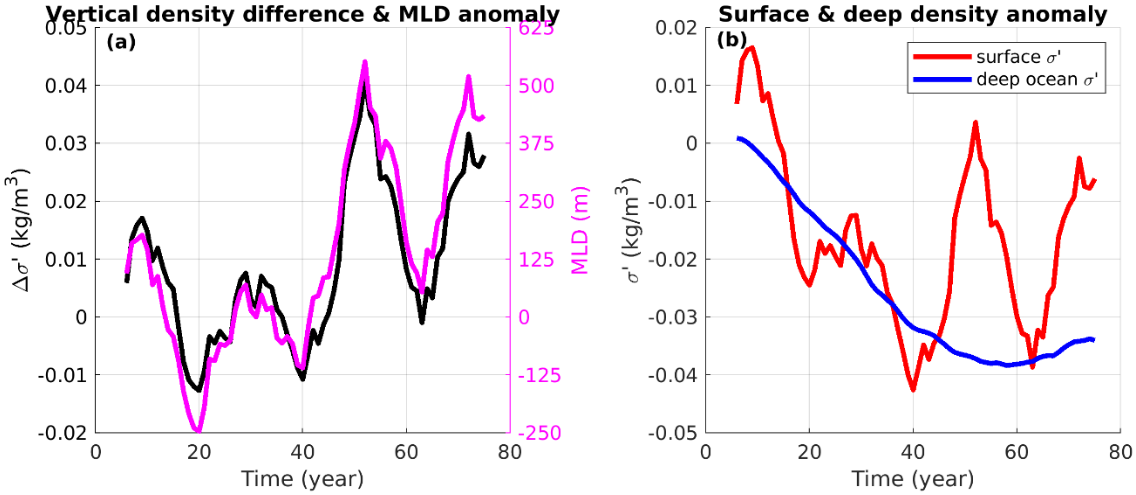

Supplementary Figure 8. Relationship between the Labrador Sea vertical density difference and mixed layer depth (MLD) anomalies. (a) Time series of March $\Delta\sigma_0$ anomaly (surface potential density anomaly minus deep ocean (1500m-2500m) averaged potential density anomaly, black line) and March MLD anomaly (magenta line) over the central Labrador Sea (black box in Fig. 1d). The vertical density difference anomaly and the MLD anomaly in the central Labrador Sea show very similar changes: the strengthening of the open-ocean Labrador Sea deep convection is associated with the increase of the vertical density difference. **(b)** Time series of March surface (red line) and deep ocean (1500m-2500m) averaged (blue line) potential density σ_0 anomalies. Their difference is shown in panel **(a)**. The 11-year running mean is used.

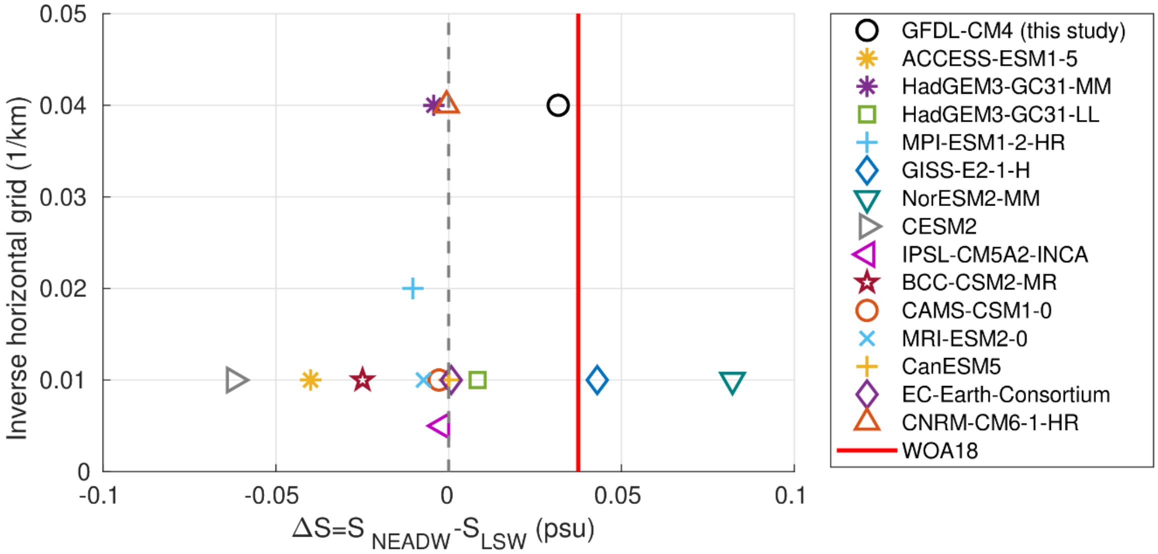

Supplementary Figure 9. Comparing the model used in this study (GFDL CM4, Methods Section) with Coupled Model Intercomparison Project Phase 6 (CMIP6) models for representing the Iceland-Scotland Overflow Water (ISOW)-associated Northeast Atlantic Deep Water (NEADW) layer in the deep Labrador Sea, evaluated by the climatological mean salinity difference between the ISOW-associated NEADW layer (2000-2500m) and the core Labrador Sea Water (LSW) layer (1000-1500m) (x-axis, $\Delta S = S_{NEADW} - S_{LSW}$) vs. the inverse horizontal grid resolution (y-axis, 1/km). All model results are averaged for the last 50 years of the corresponding control simulation. The first ensemble member (r1i1p1f1) is used for most CMIP6 models' preindustrial control simulation, except that the second ensemble member (r1i1p1f2, the only available member) is used for CNRM-CM6-1-HR's preindustrial control simulation. The observed ISOW-associated NEADW layer in the Labrador Sea is characterized by a water mass saltier than the core LSW layer above[32,51]. The red solid line represents the observed climatological mean ΔS (0.038 psu) derived from World Ocean Atlas 2018 (WOA18) data averaged over the past several decades (1955-2017). Many CMIP6 models simulate a negative or zero ΔS, indicating that the ISOW-associated NEADW layer is not represented in these models. Among the few models with a positive ΔS, the majority have a 1° (100km) coarse resolution (not enough to resolve the narrow Labrador Sea boundary current), and only the model employed in this study (GFDL CM4) has a 0.25° (25km) resolution.

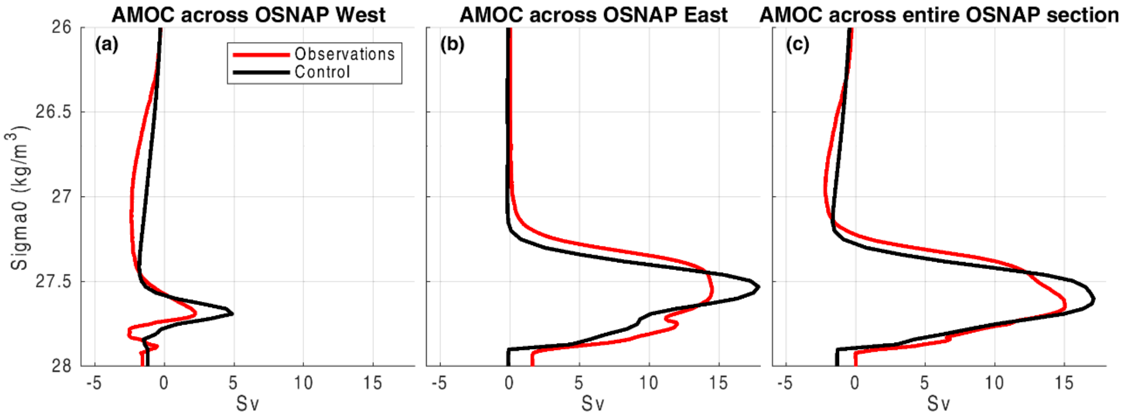

Supplementary Figure 10. Comparison of the mean Atlantic Meridional Overturning Circulation (AMOC) streamfunction (Sv , $1\,Sv = 10^6\,m^3\,s^{-1}$) across the Overturning in the Subpolar North Atlantic Program (OSNAP) section in the control simulation with OSNAP observations in density space. (a) OSNAP West. (b) OSNAP East. (c) Entire OSNAP section. Black lines: the last 40-year average of the ensemble of the control simulation. Red lines: the OSNAP observations averaged from 2014 to 2020.
